# Wanshi Shachong Xiaoji Pills Alleviate Functional Dyspepsia in Mice and Exhibit Lipid-Lowering Effects in a Hepatocyte Steatosis Model

**DOI:** 10.3390/ph19030448

**Published:** 2026-03-10

**Authors:** Xiaoyue Wang, Xinrong Ren, Rui Zhao, Junming Tu, Minghui Wang, Fanfan Wang, Yuanyuan Duan, Tao Tang, Wuxian Zhou, Qingfang Wang, Jingmao You

**Affiliations:** 1 Key Laboratory of Biology and Cultivation of Chinese Herbal Medicines, Ministry of Agriculture and Rural Affairs, Institute of Chinese Herbal Medicines, Hubei Academy of Agricultural Sciences, Enshi 445000, China; 2 Hubei Engineering Research Center of Good Agricultural Practices (GAP) Production for Chinese Herbal Medicines, Institute of Chinese Herbal Medicines, Hubei Academy of Agricultural Sciences, Enshi 445000, China; 3 Huanggang Academy of Agricultural Sciences, Huanggang 438000, China

**Keywords:** Wanshi Shachong Xiaoji Pills, functional dyspepsia, hepatocyte steatosis, safety evaluation, Gut microbiota, network pharmacology

## Abstract

**Objective**: To investigate the therapeutic effects and safety profile of Wanshi Shachong Xiaoji Pills (WSXPs) on a functional dyspepsia (FD) mouse model and to preliminarily explore its potential mechanism and impact on associated hepatic metabolism. **Methods**: An FD model was established in mice using L-arginine. Gastrointestinal motility was assessed by measuring gastric emptying and intestinal propulsion rates. Serum levels of gastrointestinal hormones (MTL, GAS, VIP, CCK) and gut microbiota composition were analyzed. A one-month repeated-dose toxicity study was conducted in normal mice to evaluate safety. The effects of WSXPs on lipid metabolism and inflammation were further examined in a hepatocyte steatosis model in vitro, and network pharmacology was employed to predict potential mechanisms. **Results**: WSXPs significantly alleviated FD symptoms by improving gastrointestinal motility, bidirectionally regulating gut hormone levels, and increasing the abundance of beneficial bacteria (*Akkermansia muciniphila*). Long-term administration showed no significant toxicity. In vitro, WSXPS reduced lipid accumulation and inflammation in hepatocytes. Network analysis identified the PI3K-Akt signaling pathway as a potentially central common target, providing a hypothesis for future mechanistic studies. **Conclusions**: WSXPs effectively improve FD symptoms, modulates gut microbiota, and exhibits potential benefits on hepatic lipid metabolism in vitro, possibly via the PI3K-Akt pathway. This hepatocyte-level finding, combined with its in vivo efficacy in FD, suggests a promising avenue for future research into its potential applications in metabolic-associated conditions. This study provides a scientific foundation for the further development and clinical application of WSXPs in treating FD.

## 1. Introduction

Obesity has become a major global public health challenge, with its associated health consequences increasingly being a primary focus of concern [1]. Digestive system dysfunction is a common comorbidity of obesity, notably represented by Functional Dyspepsia (FD) [2] and Non-Alcoholic Fatty Liver Disease (NAFLD) [3]. Recent epidemiological and clinical studies have identified a significant comorbidity between FD and NAFLD, with the prevalence of FD being markedly higher in NAFLD patients compared to NAFLD populations [4]. This frequent co-occurrence suggests a potential shared pathophysiological basis beyond simple coincidence.

In fact, the development and progression of FD and NAFLD are influenced by several common factors. First, key modifiable risk factors, such as obesity, unhealthy dietary patterns, and psychological stress, are known to contribute concurrently to both conditions [4,5]. Among these, excessive fat intake or a high-calorie diet serves as a shared trigger for both dyspeptic symptoms and NAFLD [6,7]. At the mechanistic level, studies have shown that a high-fat diet can directly induce gastrointestinal damage in mice, leading to intestinal atrophy and impairment of gastric parietal cells [8,9]. Simultaneously, it promotes the progression of NAFLD by enhancing the hepatic uptake of fatty acids, thereby exacerbating fat accumulation in the liver [10]. Emerging evidence further suggests that these external factors may act through disruption of the gut–liver axis. Dysregulation of this axis, including alterations in gut microbiota and increased intestinal permeability, is now considered a key shared pathway contributing to both visceral hypersensitivity in FD and liver injury in NAFLD [4,11]. Thus, FD and NAFLD not only frequently co-occur in clinical settings but are also closely intertwined in their etiology and underlying mechanisms. This complex pathological relationship presents both challenges and opportunities for therapeutic intervention. With its holistic approach and emphasis on syndrome differentiation, Traditional Chinese Medicine (TCM) offers considerable potential for managing complex, multi-system disorders such as the FD-NAFLD dyad.

Wanshi Shachong Xiaoji Pills (WSXPs) is a compound formulation composed of ten Chinese herbal medicines: *Codonopsis radix* (Dangshen), *Atractylodis rhizoma* (Cangzhu), *Hordei fructus germinatus* (Maiya), *Amomi fructus* (Sharen), *Quisqualis fructus* (Shijunzi), *Omphalia lapidescens schroet* (Leiwan), *Crataegi fructus* (Shanzha), *Magnoliae officinalis cortex* (Houpo), *Picrorhizae rhizoma* (Huhuanglian), and *Aloe* (Lvhui). Originally developed by Mr. Jinjie Shao, a renowned grassroots Traditional Chinese Medicine (TCM) practitioner and National Heritage Inheritor, this formula has been refined into a standardized fixed prescription through long-term clinical practice. WSXPs are indicated for infantile malnutrition (Gan syndrome) and digestive stagnation (“Jizhi”), which results from spleen–stomach deficiency syndrome [12,13]. Notably, the TCM theory of “Liver-Spleen Disharmony” provides a conceptual framework linking digestive disorders (governed by the Spleen/Stomach) to metabolic and stagnation pathologies (associated with the Liver). This theoretical alignment positions WSXPs as a compelling subject for scientific investigation into a unified therapeutic strategy for FD and NAFLD.

Modern pharmacological studies have begun to validate the biological activities of key herbs in WSXPs. Among its constituents, *Crataegi fructus*, *Codonopsis radix, Amomi fructus*, and *Hordei fructus germinatus* are recognized as homology of medicine and food by the National Health Commission of China. *Crataegi fructus* has demonstrated efficacy in the treatment of both FD and obesity [14] and has been shown to reduce lipid accumulation in high-fat, high-cholesterol diet-fed rats [1,2,3,4,5,6,7,8,9,10,11,15]. *Codonopsis radix* improves lipid metabolism disorders in high-fat, high-sugar diet-induced T2DM mice via the IRS1/PI3K/AKT signaling pathway [16], and exerts protective effects on the gastrointestinal system, including alleviation of inflammatory bowel disease, gastric ulcer relief, and hepatoprotective activity [17]; *Amomi fructus* is widely used in the treatment of gastrointestinal disorders and has shown clinical efficacy in functional dyspepsia, gastritis, and pediatric Helicobacter pylori infection [18]. Furthermore, the Maillard reaction products of *Hordei fructus germinatusdede* are considered principal active components in the treatment of FD [19].

Additional constituents of WSXPs also exhibit relevant pharmacological properties. For instance, *Magnoliae officinalis cortex* has been reported to regulate gastrointestinal hormones, modulate substance metabolism, and protect the intestinal barrier [20]. Its major bioactive compound, honokiol, has been shown to alleviate disorders of lipid and bile acid metabolism in a methionine-choline-deficient diet mouse model, while also upregulating the abundance of intestinal probiotics [21]. Importantly, a clinical extract of magnolia bark has demonstrated efficacy in reducing liver fat content in patients with NAFLD [22]. These findings provide a scientific basis for the multi-target potential of the WSXP formula.

Therefore, this study aimed to investigate the therapeutic mechanism of WSXPs against FD and its potential effects on NAFLD pathology. We employed an integrated strategy utilizing animal models, cellular assays, and network pharmacology. The efficacy of WSXPs was systematically evaluated in an FD mouse model. The direct impact on lipid metabolism and inflammation was examined in a cellular model of hepatic steatosis. Furthermore, network pharmacology analysis was applied to predict the bioactive compounds and key pathways involved. This work provides experimental evidence supporting the clinical translation and development of WSXPs as an integrated therapeutic agent.

## 2. Results

### 2.1. Safety Evaluation of Wanshi Shachong Xiaoji Pills (WSXPs)

An initial safety assessment of WSXPs was conducted following the “Technical guidelines for repeated drug administration toxicity testing" (Center for Drug Evaluation of NMPA, China, 20140513)” (China Center for Drug Evaluation, 2014) [23]. Kunming (KM) mice were administered low (WSXP-L) or medium (WSXP-M) doses of the formula via oral gavage daily for one month. Serum biochemical analysis and histopathological examination of major organs were performed to evaluate potential systemic toxicity.

**Figure 1:**
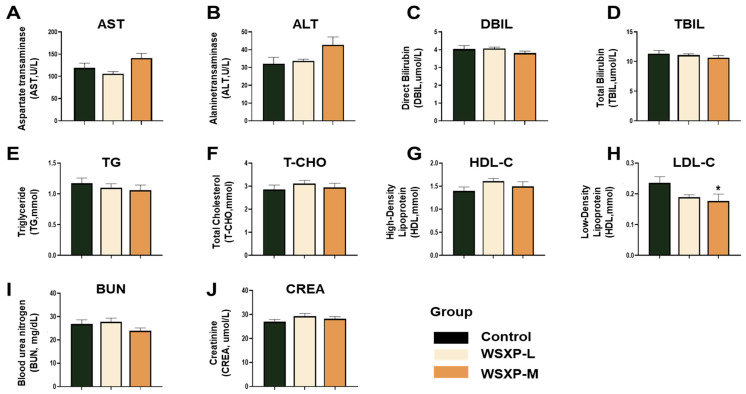
Biochemical profiling of mice following one-month consecutive administration of WSXPs. (**A**–**D**) Liver function, (**E**,**F**) kidney function, and (**G**–**J**) lipid metabolism. (**A**) Aspartate aminotransferase (AST). (**B**) Alanine aminotransferase (ALT). (**C**) Direct bilirubin (DBIL). (**D**) Total bilirubin (TBIL). (**E**) Triglycerides (TG). (**F**) Total cholesterol (T-CHO). (**G**) High-density lipoprotein cholesterol (HDL-C). (**H**) Low-density lipoprotein cholesterol (LDL-C). (**I**) Blood urea (BUN). (**J**) Creatinine (CREA). Data are presented as the mean ± SEM (*n* = 10 mice per group). * *p* < 0.05, versus the control group.

Treatment with WSXP-L and WSXP-M did not induce significant alterations in markers of liver function (ALT, AST, TBIL, DBIL; Figure 1A–D), kidney function (BUN, CREA; Figure 1I,J), or lipid metabolism (TG, T-CHO, HDL-C, LDL-C; Figure 1E–H). Notably, administration of the medium-dose WSXP (WSXP-M) specifically and significantly reduced serum LDL-C levels compared to the control group (Figure 1H, *p* < 0.05), suggesting a potential role for the formulation in modulating lipid metabolism.

**Figure 2:**
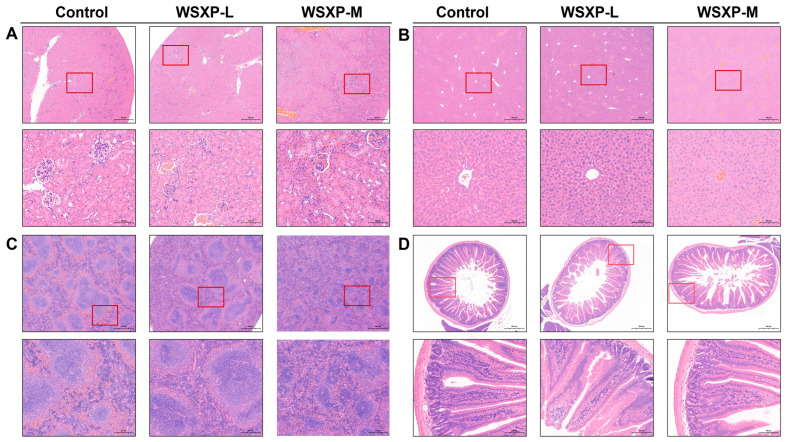
Representative H&E-stained images of liver, kidney, spleen, and small intestine from mice after long-term WSXP administration. (**A**) Kidney, (**B**) liver, (**C**) spleen, and (**D**) small intestine sections from control and WSXP-treated mice (dosages: WSXP-L, 3 g/mL; WSXP-L, 6 g/mL) are shown. The images are representative of each group (*n* = 3 per group).

H&E staining revealed no obvious pathological changes in the liver, kidney, or small intestine across all treatment groups (Figure 2). Notably, while the WSXP-L group showed no significant morphological alterations in any examined organ, the WSXP-M group exhibited a reduction in the number of splenic nodules and disorganization of the interface between the red and white pulp compared to the normal control. This finding suggests that medium-dose administration may exert an effect on splenic morphology. Furthermore, the ratios of organ-to-brain or body weight for other major organs showed no significant differences between any WSXP-treated group and the control group (Figure S1). These results indicate that WSXP-L shows a favorable safety profile across all examined parameters. For WSXP-M, while most safety indicators remained normal, the observed splenic morphological changes suggest that medium-dose administration may exert an effect on splenic morphology, which merits further investigation.

### 2.2. WSXP Ameliorates Gastrointestinal Dysfunction in an FD Mouse Model

**Figure 3:**
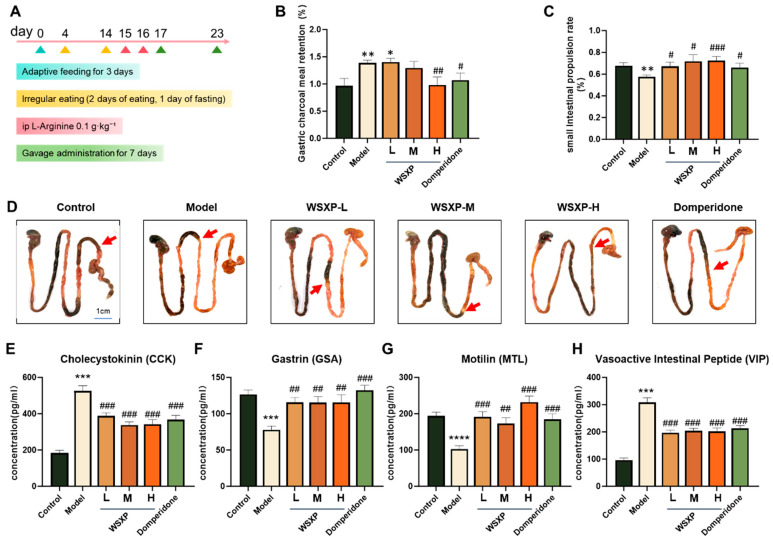
Effects of WSXPs on gastrointestinal motility in an FD model. (**A**) Schematic diagram illustrating the experimental timeline for FD model establishment and drug intervention. (**B**) Gastric emptying function assessed by gastric charcoal meal retention rate. (**C**) Small intestinal propulsion rate, measured as the percentage of distance traveled by the charcoal meal relative to the total length of the small intestine. (**D**) Representative pictures of gastrointestinal transit of the charcoal meal, the red arrow indicates the endpoint of the charcoal meal movement in the small intestine. (**E**–**H**) Serum levels of key gastrointestinal hormones measured by ELISA: (**E**) Cholecystokinin (CCK), (**F**) Gastrin (GAS), (**G**) Motilin (MTL), and (**H**) Vasoactive Intestinal Peptide (VIP). Data are presented as the mean ± SEM (*n* = 10 mice per group). * *p* < 0.05, ** *p* < 0.01, *** *p* < 0.001, **** *p* < 0.0001 versus the normal control group. # *p* < 0.05, ## *p* < 0.01, ### *p* < 0.001 versus the model group.

A functional dyspepsia (FD) model was successfully established in Kunming mice through a combination of irregular feeding and L-arginine (L-Arg) administration (Figure 3A). Following one week of post-modeling drug intervention, significant gastrointestinal motility disorders were observed in the model group compared to the normal control. Specifically, the model group exhibited a 43.8% increase in gastric charcoal meal retention (*p* < 0.01, Figure 3B) and a 15% decrease in small intestinal propulsion rate (*p* < 0.01, Figure 3C,D), confirming successful FD model replication.

Treatment with WSXPs effectively reversed these dysfunctions. Compared to the model group, the low (3 g/kg)-, medium (6 g/kg)-, and high (12 g/kg)-dose WSXP groups, as well as the domperidone (positive control, 5 mg/kg) group, significantly increased the small intestinal propulsion rate by 17.5%, 26.3%, 28.1%, and 15.8%, respectively (*p* < 0.001, Figure 3C,D). Furthermore, the high-dose WSXP group and the domperidone group significantly reduced gastric charcoal meal retention by 31.7% and 23.0% (*p* < 0.05, Figure 3B). These results indicate a dose-dependent improvement in gastrointestinal motility by WSXPs, with the high-dose regimen showing the most pronounced effect, comparable to that of domperidone.

To elucidate the underlying mechanism, serum levels of key gastrointestinal regulatory peptides were analyzed by ELISA. The model group showed a significant decrease in motilin (MTL) and gastrin (GAS) levels by 47.4% and 38.7% (*p* < 0.01, Figure 3F,G), alongside a significant increase in vasoactive intestinal peptide (VIP) and cholecystokinin (CCK) levels by 68.9% and 65.2% (*p* < 0.001, Figure 3E,H). WSXP treatment reversed these aberrant hormonal changes. All WSXP-treated groups significantly elevated the decreased levels of MTL and GAS and significantly reduced the elevated levels of VIP and CCK compared to the model group.

### 2.3. Effects of WSXPs on the Gut Microbiota in FD Mice

**Figure 4:**
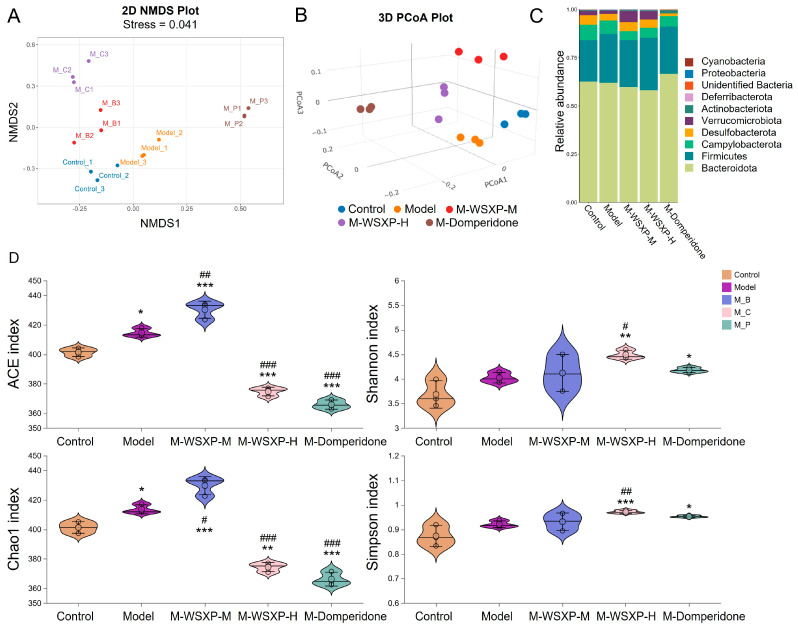
Gut microbiota modulation by WSXPs in an FD mouse model. (**A**) NMDS and (**B**) PCoA plots (Bray–Curtis distance) illustrating β-diversity among groups: Control, FD Model (M), M + WSXP-L, M + WSXP-H, and FD + Domperidone (M-P). (**C**) Taxonomic profile showing the relative abundance of the top 10 genera. (**D**) α-Diversity indices (Chao1 and Shannon). Data were obtained from pooled samples (*n* = 10 mice per group, pooled and sequenced in triplicate); * *p* < 0.05, ** *p* < 0.01, *** *p* < 0.001 versus the normal control group. # *p* < 0.05, ## *p* < 0.01, ### *p* < 0.001 versus the model group.

The impact of WSXPs on the gut microbiota in FD mice was assessed by analyzing both beta and alpha diversity. Principal Coordinates Analysis (PCoA) and Non-metric Multidimensional Scaling (NMDS) revealed a clear separation between the samples from the FD model group and those from the normal control group (Figure 4A,B), indicating that FD induction significantly altered the overall structure of the gut microbiota. In terms of alpha diversity, the FD model group exhibited significant increases in species richness indices (ACE and Chao1) and composite diversity indices (Shannon and Simpson) compared to the control group (Figure 4D).

Analysis of microbial composition at the phylum level showed that the relative abundances of several gut health-associated *phyla* were decreased in the FD model group. Specifically, the abundances of *Verrucomicrobiota* (a *phylum* associated with intestinal barrier function) and *Actinobacteriota* were lower compared to the control group (Figure 4B).

**Figure 5:**
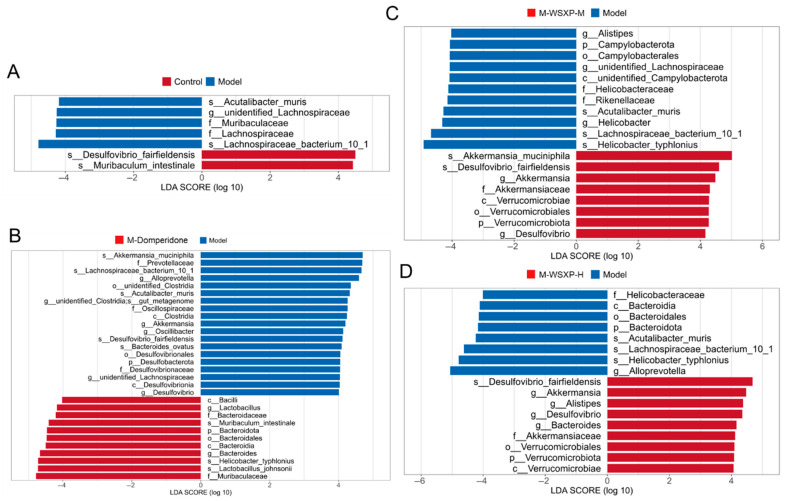
Identification of differentially abundant bacterial biomarkers by LEfSe analysis. (**A**) FD model group versus the control group. (**B**) Positive drug group versus the Model group. (**C**) WSXP-medium dose group versus the Model group; (**D**) WSXP-high dose group versus the Model group. |Log LDA score| > 4, top 30 biomarkers shown.

To investigate the regulatory effects of different interventions on the FD, Linear Discriminant Analysis Effect Size (LEfSe) analysis was performed. The results demonstrated that both medium- and high-dose WSXP interventions specifically enriched the probiotic *Akkermansia muciniphila*. This enrichment was observed across its entire *taxonomic* lineage, showing significant increases from the phylum level (*p_Verrucomicrobiota*) down to the *genus* level (*g_Akkermansia*) (Figure 5). These findings suggest that WSXPs may ameliorate FD by specifically promoting this key beneficial bacterium known for its mucosal repair functions.

### 2.4. WSXP Attenuates Lipid Accumulation and Inflammation in a HepG2 Cell Model of Non-Alcoholic Fatty Liver (NAFLD)

Based on the significant inhibitory effect on serum LDL-C observed in the chronic toxicity study, we further investigated the potential lipid-lowering and anti-inflammatory efficacy of WSXPs at the cellular level. A model of NAFLD was established in HepG2 cells by co-induction with sodium oleate and sodium palmitate to evaluate the effects of WSXPs on intracellular lipid accumulation and related inflammatory responses.

**Figure 6:**
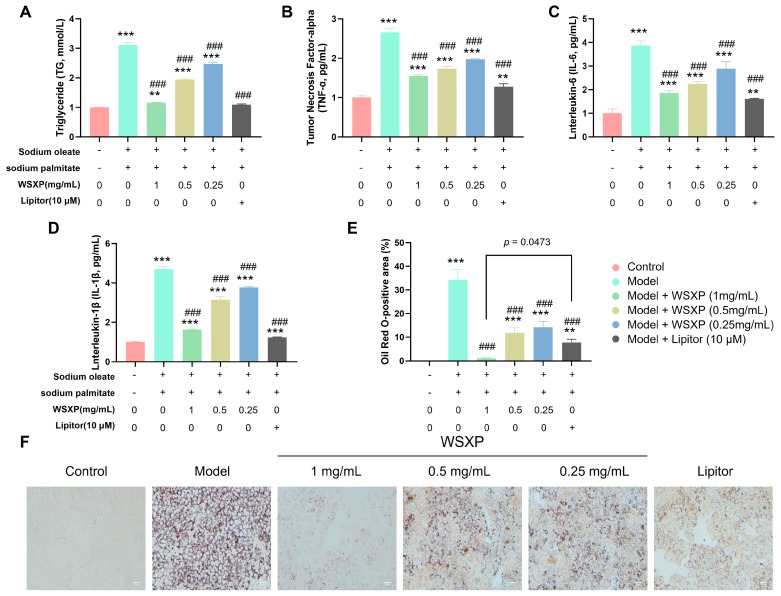
Effects of WSXPs on lipid metabolism and inflammatory response in HepG2 cell line. (**A**) Triglyceride (TG) content in cell culture supernatant measured by enzymatic assay. (**B**–**D**) Secretion of pro-inflammatory cytokines: (**B**) TNF-α, (**C**) IL-6, and (**D**) IL-1β, quantified by ELISA. (**E**) Quantification of intracellular lipid accumulation assessed by Oil Red O staining area (% of total cell area). (**F**) Representative micrographs of Oil Red O staining, scale bars: 50 μm. Data are presented as mean ± SEM (*n* = 3 independent experiments with triplicate samples). ** *p* < 0.01, *** *p* < 0.001 vs. control group, ### *p* < 0.001 vs. Model group.

The results demonstrated that co-induction significantly elevated the levels of triglycerides (TG) and the inflammatory cytokines TNF-α, IL-6, and IL-1β in the cell culture supernatant of the model group compared to the control. Intervention with WSXPs at concentrations ranging from 1 mg/mL to 0.25 mg/mL effectively suppressed the accumulation of TG and the release of these inflammatory factors in a dose-dependent manner (Figure 6A–D, all *p* < 0.001 vs. model group).

The Oil Red O staining results were consistent with the biochemical findings. Microscopic observation revealed substantial red-stained lipid droplets in the model group cells, which were markedly reduced in all WSXP-treated groups. Notably, treatment with 1 mg/mL WSXP significantly decreased the Oil Red O-positive area within the cells, and this effect was superior to that observed in the atorvastatin (Lipitor) positive control group.

These findings confirm that WSXP possesses significant dual efficacy in reducing lipid accumulation and suppressing inflammation in this cellular model, providing direct in vitro evidence supporting the lipid-lowering effects observed in the animal experiments.

### 2.5. Network Pharmacology Analysis Identifies the Potential Material Basis and Mechanism of Action for WSXPs

**Figure 7:**
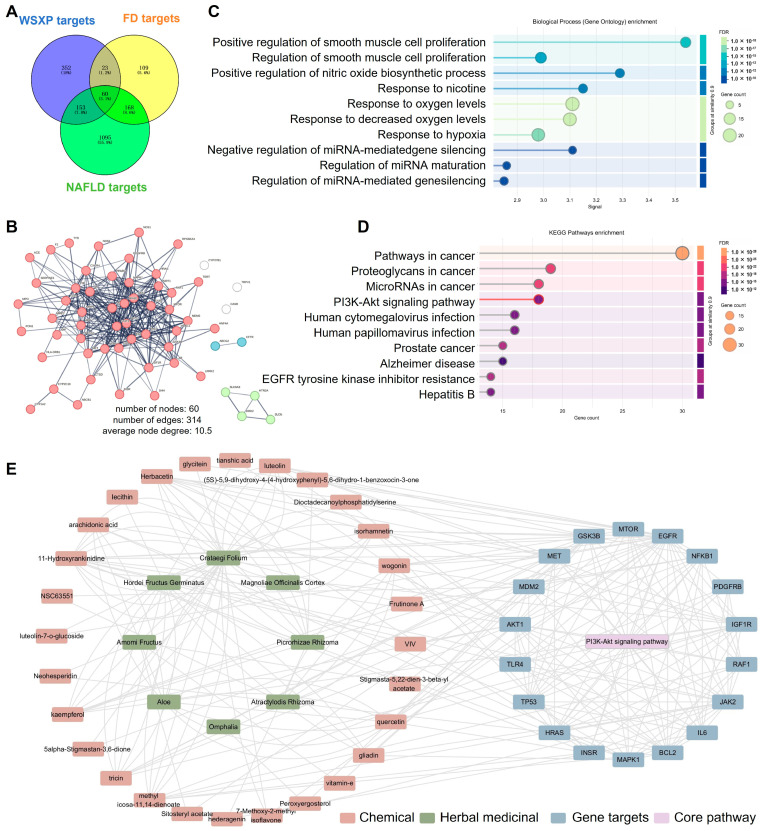
Network pharmacology-based prediction of targets and mechanisms of WSXPs against FD and NAFLD. (**A**) Venn diagram showing the intersection of predicted WSXP compound targets with FD- and NAFLD-related disease targets. (**B**) Protein–protein interaction (PPI) network of the intersecting core targets. (**C**) Gene Ontology (GO) enrichment analysis of the core targets. (**D**) Kyoto Encyclopedia of Genes and Genomes (KEGG) pathway enrichment analysis of the core targets. (**E**) Integrated “Disease-Target-Pathway” interaction network diagram.

Network pharmacology was employed to predict the active components and mechanisms of WSXPs against FD and NAFLD. Screening identified 83 bioactive compounds from WSXPs, corresponding to 588 predicted targets. These targets intersected with 360 FD-related and 1476 NAFLD-related genes, yielding 60 core potential therapeutic targets (Figure 7A). A protein–protein interaction network of these 60 targets (STRING confidence > 0.7) showed strong connectivity (60 nodes, 314 edges, Figure 7B). GO analysis revealed enrichment in biological processes critical for gastrointestinal function, including the regulation of smooth muscle cell proliferation and the response to oxygen levels (Figure 7C).

KEGG pathway enrichment analysis showed that the core targets were predominantly involved in the PI3K-Akt signaling pathway, a central regulator of cell metabolism, growth, and motility. A comprehensive “Compound-Target-Pathway” network was subsequently constructed, revealing that 28 key bioactive compounds derived from multiple herbal constituents—including *Omphalia*, *Codonopsis Radix*, *Atractylodis Rhizoma*, *Magnoliae Officinalis Cortex*, *Amomi Fructus*, *Crataegi Folium*, *Hordei Fructus Germinatus*, and *Picrorhizae Rhizoma*—were implicated in the modulation of this central pathway. These results suggest specific targets and compounds that may contribute to the formula’s therapeutic effects, providing a basis for future experimental validation.

## 3. Discussion

This integrated study evaluates the therapeutic potential and safety profile of WSXPs for Functional Dyspepsia (FD) and Non-Alcoholic Fatty Liver Disease (NAFLD). For the FD component, an animal model was established using an irregular diet combined with intraperitoneal injection of L-arginine (L-Arg). This modeling approach aligns closely with the clinical etiology of FD, as irregular eating habits are a key factor in the development of this condition [14]. The mechanism by which L-Arg induces FD is primarily mediated through the nitric oxide (NO) pathway: L-Arg serves as a substrate for NO synthesis, and NO is a potent regulator of gastric motility and secretion, inhibiting gastric motility by activating cholinergic neurons in the vagal nerve [24]. For the NAFLD component, a HepG2-based cellular model of steatosis was employed to investigate the lipid-lowering effects of WSXPs. This in vitro model is widely recognized as a standardized and reproducible system for studying NAFLD and related metabolic disorders [25].

Experimentally, WSXP (3~12 g/kg) was shown to alleviate core symptoms in the FD model mice. It significantly increased the gastric emptying rate and intestinal propulsion rate. Concurrently, WSXPs upregulated serum levels of motilin (MTL) and gastrin (GAS) to enhance gastrointestinal contraction and motility. Conversely, it downregulated levels of vasoactive intestinal peptide (VIP) and cholecystokinin (CCK), thereby mitigating their physiological inhibitory effects on gut movement. This bidirectional regulatory effect demonstrates its multi-target action. Furthermore, in a chronic toxicity study, WSXP-M (6 g/kg) specifically reduced serum low-density lipoprotein cholesterol (LDL-C) levels (*p* < 0.05). In vitro, WSXP (1 mg/mL) was confirmed to inhibit lipid production in a cellular model.

Our investigation into WSXP’s effects on FD revealed that it significantly increased the relative abundance of the beneficial bacterial phylum *Verrucomicrobiota* in the gut of model mice. This phylum’s representative species, *Akkermansia muciniphila*, is strongly associated with improved intestinal barrier function [26]. Mechanistically, *A. muciniphila* produces short-chain fatty acids (SCFAs) like acetate, propionate, and butyrate by degrading gastrointestinal mucins [27,28]. These SCFAs act as signaling molecules that regulate host lipid metabolism [29] and are crucial for energy balance and glucose homeostasis [30]. They also modulate neurotransmitter levels, enhancing gut 5-HT synthesis and reducing SERT expression, which improves gut–brain axis communication [31]. Furthermore, supplementation with *A. muciniphila* promotes the proliferation of intestinal stem cells, increases goblet cell numbers, and enhances epithelial regeneration [32]. These findings suggest that WSXPs may ameliorate FD pathology, in part, by modulating the abundance of *Verrucomicrobiota* and leveraging the multifaceted beneficial roles of *A. muciniphila*.

The observed hepatic benefits of WSXPs in vitro, coupled with network pharmacology predictions, point to the PI3K-Akt signaling pathway as a convergent mechanism. This pathway centrally regulates cell growth, metabolism, and inflammation. In the liver, PI3K-Akt activation can promote lipogenesis via SREBP-1c [33], while its modulation is also implicated in the action of NAFLD therapeutics like Saroglitazar [34]. The modulation of the PI3K/Akt/NF-κB pathway has been shown to alleviate Helicobacter pylori-induced gastritis [35]. *H. pylori* infection is also a recognized contributing factor to FD [4]. Though these findings are predictive and require experimental validation, they identify the PI3K-Akt pathway as a candidate mechanistic target for future exploration of WSXPs in ameliorating both hepatic steatosis and gastrointestinal dysfunction.

While this integrated study provides compelling evidence for the therapeutic potential of WSXPs, several inherent limitations should be acknowledged to contextualize the findings and guide future work. First, the HepG2 cell line used for the NAFLD component is derived from a human hepatoma, which, despite being a widely recognized and standardized in vitro model for studying hepatic steatosis [25], does not fully recapitulate the metabolic characteristics of primary human hepatocytes. This limits the translational potential of the lipid-lowering effects observed. Second, although high-fat intake is a common etiological factor shared by both FD and NAFLD, the experimental conditions in this study did not maintain a consistent high-fat induction paradigm across the in vivo FD model and the in vitro steatosis model. The FD model was established using an irregular diet combined with L-arginine injection, while the hepatic steatosis model was induced by oleic acid in HepG2 cells. Consequently, despite the significant therapeutic effects observed in both models, the consistency of WSXP’s efficacy against FD-NAFLD comorbidity should be interpreted with caution. The current findings demonstrate that WSXP alleviates FD symptoms in vivo and reduces fatty acid-induced lipid accumulation in hepatocytes in vitro, highlighting its potential dual therapeutic value. However, whether these effects are directly transferable to a comorbid condition remains to be validated. Third, the key signaling pathways, such as PI3K-Akt, were primarily identified through network pharmacology prediction. Subsequent studies are imperative to experimentally validate these computational predictions and elucidate the precise mechanistic underpinnings. Future research must prioritize the development and application of a validated comorbid animal model that recapitulates both FD and NAFLD features under consistent dietary conditions to directly evaluate the holistic efficacy of WSXPs.

## 4. Materials and Methods

### 4.1. Reagents and Drug

*Wanshi Shachong Xiaoji* Pills (WSXPs) were provided by Luotian Wanmizhai Hospital (Batch No.: 202504002, Hubei, China). The pills is composed of the following crude herbs: *Codonopsis Radix* (Dangshen, 8 g), *Atractylodis Rhizoma* (Cangzhu, 5 g), *Crataegi Fructus* (Shanzha, 8 g), *Hordei Fructus Germinatus* (Maiya, 10 g), *Amomi Fructus* (Sharen, 6 g), *Quisqualis Fructus* (Shijunzi, 5 g), *Omphalia* (Leiwan, 5 g), *Magnoliae Officinalis Cortex* (Houpo, 5 g), *Picrorhizae Rhizoma* (Huhuanglian, 2 g), and *Aloe* (Lvhui, 1 g). The crude herbs were mixed according to the specified formula ratio and decocted with water three times. The three decoctions were combined, heated to concentrate, and dried to obtain the extract. From one formula (55 g crude herbs), approximately 20 g of concentrated pills were produced, corresponding to a crude drug ratio of 2.75 g crude herb per gram of pills.

Domperidone tablets (Batch No.: 20250101) were purchased from Shanxi Baotai Pharmaceutical Co., Ltd. (Shanxi, China). Reagents for the in vivo study included L-Arginine (Cat# ST1429-50g, Beyotime, Shanghai, China), sodium carboxymethyl cellulose (Cat# A360250526, Beyotime, Shanghai, China), skimmed milk powder (Cat# A355A250618, Beyotime, Shanghai, China), 1.25% tribromoethanol (Cat# JT0781, Beijing Jitian Biological Technology, Beijing, China), general tissue fixative (Cat# GP25023040072, Servicebio, Wuhan, China), phosphate-buffered saline (PBS, Cat# 17L01A32, Boster, Wuhan, China), glucose (Batch No.: 20240516, Tianjin Bodi Chemical, Tianjin, China), soluble starch (Batch No.: 20181210, Sinopharm, Shanghai China), and activated charcoal (Cat# 1106G051, Solarbio, Wuhan, China). For the in vitro experiments, Dulbecco’s Modified Eagle Medium (DMEM, Cat# PM150210, Procell, Wuhan, China), Fetal Bovine Serum (FBS, BC-SE-FBS01, Sbjbio life Science, Nanjing, China), and a fatty acid mixture of sodium oleate and sodium palmitate (Cat# C3461, Solarbio, Wuhan, China) were used. An Oil Red O staining kit (Cat# C0157S, Beyotime, Shanghai, China) was employed for lipid droplet visualization. Enzyme-linked immunosorbent assay (ELISA) kits for detecting mouse Motilin (MTL, Cat# MU30329), Gastrin (Cat# MU30444), Cholecystokinin (CCK, Cat# MU30355), and Vasoactive Intestinal Peptide (VIP, Cat# MU30064) were sourced from Wuhan Bionline Biotechnology (Wuhan, China). Additionally, ELISA kits for quantifying Triglycerides (TG, Cat# E-BC-K261-M), Tumor Necrosis Factor-alpha (TNF-α, Cat# E-EL-H0109), Interleukin-6 (IL-6, Cat# E-EL-H61), and Interleukin-1β (IL-1β, Cat# E-EL-H0149) in cell culture supernatant were obtained from Elabscience (Wuhan, China).

### 4.2. Grouping and Treatment of Animals

A total of 90 male KM mice (20 ± 2 g, 6–8 weeks old) were obtained from Wuhan Myhalic Biotechnological Co., Ltd. (Wuhan, China, SCXK2025-0018). All animals were housed under specific pathogen-free conditions at 22 ± 2 °C with 50–60% relative humidity and a 12 h light/dark cycle, with free access to standard chow and water. Cages were cleaned weekly, and animal health and behavior were monitored daily throughout the experiment. After 5 days of acclimatization, mice were stratified by body weight and sequentially numbered. Using a random number table, mice within each weight stratum were randomly assigned to the experimental groups. For the drug efficacy study, 60 mice were randomly assigned to six groups (n = 10 per group): (1) Control group; (2) FD model group; (3) WSXP low-dose group (3 g/kg); (4) WSXP medium-dose group (6 g/kg); (5) WSXP high-dose group (12 g/kg); and (6) Domperidone group (5 mg/kg; positive control). The FD model was established in all groups except the control group using an irregular feeding regimen, which involved alternating between 2 days of normal feeding and 1 day of fasting for a total duration of 10 days. After this modeling period, the mice received their designated drug treatments for 7 days. The FD mouse model was established by intraperitoneal injection of L-arginine, a well-established method for inducing gastrointestinal motility disorders characterized by delayed gastric emptying [14].

For the toxicological assessment, the remaining 30 mice were randomly divided into three groups (n = 10 per group): (1) Control group; (2) WSXP medium-dose group (6 g/kg); and (3) WSXP high-dose group (12 g/kg).

### 4.3. Detection of Gastric Emptying Rate and Small Intestinal Motility Rate in Mice

Gastrointestinal motility was evaluated by measuring gastric emptying and intestinal propulsion rates. Briefly, the paste was formulated by mixing 5 g of carboxymethyl cellulose, 8 g of milk powder, 4 g of sucrose, 4 g of soluble starch, and 1 g of activated charcoal with 128 mL of distilled water, yielding approximately 150 g of a uniform semi-solid mixture. Each mouse was gavaged with 0.5 mL of the paste. After 30 min, the mice were anesthetized and euthanized. The entire stomach was then excised and weighed (total weight). Subsequently, the stomach was incised along the greater curvature, and its contents were rinsed out with PBS buffer. The emptied stomach was blotted dry on filter paper and weighed again (net weight). The small intestine (from the pylorus to the cecum) was carefully separated, laid flat on a measuring plate, and photographed for analysis. The gastric emptying rate (GER) and intestinal propulsion rate (IPR) were calculated as follows:

GER (%) = [(Total stomach weight − Net stomach weight)/0.5] × 100%

IPR (%) = (Distance traveled by the charcoal/Total length of the small intestine) × 100%

### 4.4. 16S rRNA Analysis of Mouse Cecal Contents

After euthanasia, cecal contents were collected from each mouse (n = 10 per group). To obtain a representative sample for sequencing, equal amounts of DNA from all 10 mice within the same group were pooled to construct a single library per group. The pooled libraries were then sequenced in triplicate on the Illumina NovaSeq 6000 platform (Illumina, San Diego, CA, USA) using paired-end sequencing. All raw sequencing data were processed and analyzed using the online platform CloudPlatform (https://cloud.metware.cn/, 15 October 2025).

### 4.5. Serum Level of MTL, GAS, VIP, CCK

Serum levels of gastrointestinal hormones, including Motilin (MTL), Gastrin (GAS), Vasoactive Intestinal Peptide (VIP), and Cholecystokinin (CCK), were measured using commercial enzyme-linked immunosorbent assay (ELISA) kits. All procedures were strictly performed according to the manufacturers’ protocols.

### 4.6. WSXP Safety Evaluation

Following the “Technical Guidelines for Repeated Dose Toxicity Testing of Drugs,” the control group and groups receiving medium- and high-dose WSXPs were treated for one month. Serum was subsequently collected to assess liver function (ALT, AST, TBIL, DBIL, DBIL/TBIL ratio), renal function (UREA, CREA), and lipid profiles (TG, T-CHO, HDL, LDL). All biochemical analyses were performed using a fully automated biochemical analyzer (Chemray 240, Rayto Life and Analytical Sciences Co., Ltd., Shenzhen, China). Organ tissues were immediately fixed in 4% paraformaldehyde for 72 h for subsequent pathological sectioning and staining.

### 4.7. Cell Culture and Treatment

#### 4.7.1. Cell Culture

HepG2 cells were obtained from FuHeng Biology (Shanghai, China). Cells were cultured in DMEM supplemented with 10% fetal bovine serum (FBS) and 1% penicillin-streptomycin solution. Cells were maintained at 37 °C in a humidified atmosphere containing 5% CO_2_ and subcultured upon reaching 80–90% confluence.

#### 4.7.2. Establishment of the Nonalcoholic Fatty Liver Cell Model

To induce intracellular lipid accumulation, HepG2 cells were exposed to the final induction medium containing 500 μM sodium oleate and 250 μM sodium palmitate for 48 h to establish the in vitro nonalcoholic fatty liver disease (NAFLD) model.

#### 4.7.3. Experimental Grouping and Drug Treatment

After model induction, cells were assigned to the following groups: (1) Control group: cells cultured in normal medium. (2) Model group: cells treated with the fatty acid mixture only. (3) WSXP-treated groups: model cells co-treated with WSXPs at final concentrations of 1.0 mg/mL, 0.5 mg/mL, and 0.25 mg/mL. (4) Positive control group: model cells co-treated with atorvastatin calcium (10 µM). All treatments were administered for 24 h.

#### 4.7.4. Measurement of Biochemical Markers by ELISA

Following the 24 h treatment period, the culture supernatant was collected. The concentrations of triglycerides (TG), tumor necrosis factor-alpha (TNF-α), interleukin-6 (IL-6), and interleukin-1β (IL-1β) in the supernatant were quantified using commercial enzyme-linked immunosorbent assay (ELISA) kits following the manufacturer’s instructions.

#### 4.7.5. Oil Red O Staining

To visually assess intracellular lipid accumulation, cells were seeded in 6-well plates at a density of 1 × 10^5^ cells per well and subjected to the treatments described above. After treatment, cells were washed with phosphate-buffered saline (PBS), fixed with 4% paraformaldehyde for 10 min at room temperature, and then stained using an Oil Red O staining kit according to the provided protocol. For quantitative analysis of lipid accumulation, the area of Oil Red O staining was measured using ImageJ software (version: Portable).

### 4.8. Network Pharmacology Analysis

#### 4.8.1. Identification of Common Targets and Network Construction

Active ingredients in each herb of WSXP were retrieved from the Traditional Chinese Medicine Systems Pharmacology Database and Analysis Platform (TCMSP, https://www.tcmsp-e.com/, 15 June 2025). The screening criteria were set as oral bioavailability (OB) ≥ 30% and drug-likeness (DL) ≥ 0.18. Canonical SMILES of the compounds were obtained for target prediction. The putative protein targets of the screened compounds were predicted using the SwissTargetPrediction database (http://swisstargetprediction.ch/, 20 June 2025). The prediction was restricted to Homo sapiens, and targets with a probability score > 0 were collected as potential WSXP targets. Targets associated with “functional dyspepsia” and “non-alcoholic fatty liver disease” were obtained from the GeneCards database (https://www.genecards.org/, 20 December 2025). The relevance score cutoff was set to >10. The potential WSXP targets were intersected with the FD- and NAFLD-related targets using a Venn diagram to identify the common targets, which were considered the core targets for WSXPs against FD/NAFLD. The protein-protein interaction (PPI) network of these core targets was then constructed using the STRING database (https://string-db.org/, 20 December 2025), with the species set to “Homo sapiens” and a minimum required interaction score > 0.7.

#### 4.8.2. Enrichment Analysis and Visual Network Integration

Gene Ontology (GO) and Kyoto Encyclopedia of Genes and Genomes (KEGG) pathway enrichment analyses were performed on the core target set via the STRING platform. Finally, the interaction data from the STRING network, along with the compound and disease information, were imported into Cytoscape software (version: 3.10) to construct and visualize a comprehensive “Compound-Target-Pathway” network.

### 4.9. Statistical Analysis

Results are expressed as mean ± standard error of the mean (SEM). All statistical analyses were performed using GraphPad Prism software (version 8.0). Prior to analysis, the Shapiro–Wilk test was used to verify the normality of data distribution. For comparisons between two groups, an unpaired Student’s t-test was applied. For multiple group comparisons, one-way analysis of variance (ANOVA) was conducted, followed by Tukey’s post hoc test to correct for multiple comparisons and ensure the reliability of pairwise analyses. Statistical significance was defined as *p* < 0.05.

## 5. Conclusions

This study provides the first integrated evaluation of Wanshi Shachong Xiaoji Pills (WSXPs) for functional dyspepsia (FD) and non-alcoholic fatty liver disease (NAFLD). WSXPs improved gastrointestinal dysfunction in FD model mice. It regulated key gut hormones and increased beneficial gut bacteria like *Akkermansia muciniphila*. In cellular studies, WSXPs also reduced liver fat accumulation and inflammation. Network analysis suggested that the PI3K-Akt pathway might link these dual benefits. While further validation is needed, this work supports WSXPs as a promising multi-target approach for managing both FD and NAFLD.

## Data Availability

The original contributions presented in this study are included in the article. Further inquiries can be directed to the corresponding author.

## Supplementary Materials

The following supporting information can be downloaded at: https://www.mdpi.com/article/10.3390/ph19030448/s1, Figure S1. Relative organ weights expressed as ratios to brain weight (A–C) or body weight (D–F). (A) Liver/Brain. (B) Kidney/Brain. (C) Spleen/Brain. (D) Liver/Body weight. (E) Kidney/Body weight. (F) Spleen/Body weight. Data are mean ± SEM; n = 10/group.
