# Supplementary material for: Wanshi Shachong Xiaoji Pills Alleviate Functional Dyspepsia in Mice and Exhibit Lipid-Lowering Effects in a Hepatocyte Steatosis Model
Source: Pharmaceuticals (Basel). 2026 Mar 10;19(3):448. doi: 10.3390/ph19030448 (PMC13029522; doi:10.3390/ph19030448)
Supplement: Supplementary file 1 [file pharmaceuticals-19-00448-s001.zip › pharmaceuticals-4171293-supplementary.pdf]

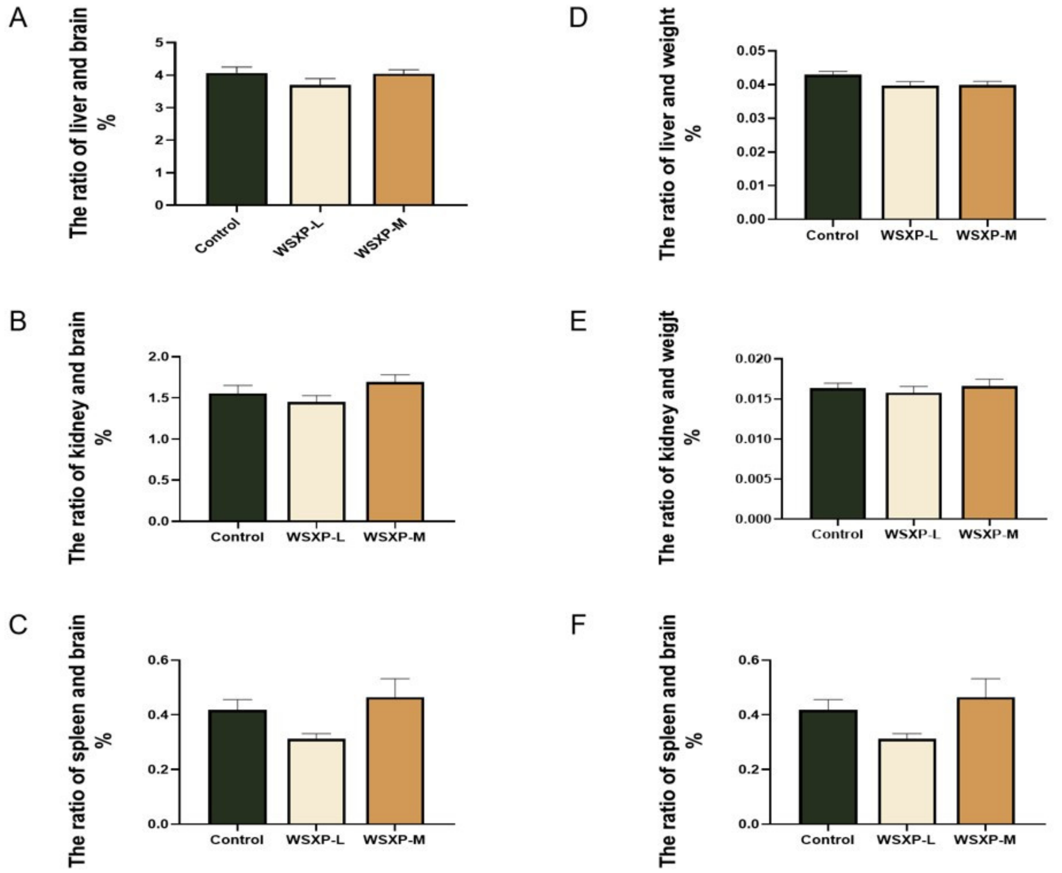

Figure S1. Relative organ weights expressed as ratios to brain weight (A-C) or body weight (D-F). (A) Liv-er/Brain. (B) Kidney/Brain. (C) Spleen/Brain. (D) Liver/Body weight. (E) Kidney/Body weight. (F) Spleen/Body weight. Data are mean ± SEM; n=10/group.
